# Operant behavioral responses to orofacial cold stimuli in rats with chronic constrictive trigeminal nerve injury: effects of menthol and capsazepine

**DOI:** 10.1186/1744-8069-9-28

**Published:** 2013-06-14

**Authors:** Xiaozhuo Zuo, Jennifer X Ling, Guang-Yin Xu, Jianguo G Gu

**Affiliations:** 1 Department of Anesthesiology and the Graduate Program in Neuroscience, The University of Cincinnati College of Medicine, PO Box 670531231 Albert Sabin Way, Cincinnati, OH 45267-0531, USA; 2 Department of Anesthesiology, Guiyang Medical University, 9 Beijing Road, Guiyang, Guizhou, 550004, PRC; 3 Institute of Neuroscience and Department of Neurobiology and Psychology, Key Laboratory of Pain Research & Therapy, Soochow University, Suzhou 215123, PRC

**Keywords:** Trigeminal neuropathic pain, TRPM8 channel, Cold allodynia and hyperalgesia, Orofacial operant behavior test, Menthol, Capsazepine

## Abstract

Both spinal and trigeminal somatosensory systems use the TRPM8 channel as a principal transducer for detecting cold stimuli. It is currently unclear whether this cold transducer may play a role in trigeminal neuropathic pain manifesting cold allodynia and hyperalgesia. In the present study, trigeminal neuropathy was induced by chronic constrictive nerve injury of the infraorbital nerve (ION-CCI). Behavioral responses to cold stimuli in orofacial regions were assessed by the newly developed orofacial operant test in the ION-CCI rats. We tested menthol and capsazepine, two compounds that can activate and inhibit TRPM8 respectively, on orofacial operant responses to cold stimuli in ION-CCI rats. Testing animals performed operant tasks by voluntarily contacting their orofacial regions to a cold stimulation module in order to access sweetened milk as a reward, and contact time and number of the operant behaviors were automatically recorded. Total contact time was significantly reduced at the cooling temperatures of 17°C and 12°C in ION-CCI group in comparison with sham group, indicating the presence of cold allodynia and hyperalgesia in ION-CCI rats. When menthol was administered to ION-CCI rats, total contact time was further reduced and total contact number increased at the cooling temperatures. In contrast, after administration of capsazepine to ION-CCI rats, total contact time was significantly increased at the cooling temperatures. The behavioral outcomes support the idea that TRPM8 plays a role in cold allodynia and hyperalgesia following chronic trigeminal nerve injury.

## Background

Trigeminal neuralgia is the most common debilitating orofacial neuropathic pain disorder [1,2] and is known to be poorly response to the treatments including opioids in human patients [3,4]. One common symptom of trigeminal neuralgia is cold allodynia and hyperalgesia [2,5], an exaggerated painful condition that is induced by innocuous cold or mild noxious cold temperatures. Mechanisms underlying the abnormal sensitivity to cold temperatures in trigeminal neuropathy have not been well studied using complex, non-reflexive behavioral assessment paradigms. Most of previous behavioral assessment methods in pre-clinical pain research measured simple reflexive responses that do not necessarily represent pain behaviors in experimental animals [6].

Sensory responses to cold stimuli are believed to be mediated by a number of molecules expressed on primary afferent neurons, including TRPM8 [7,8], TRPA1 [9], Kv1 and two-pore (K2P) potassium channels [10-12]. Among them, TRPM8 has been established to be a principal cold transducer with the capability to detect both innocuous and noxious cooling temperatures [13-15]. TRPM8 channels are expressed on a subpopulation of afferent neurons located in both somatic and trigeminal nervous systems [7,8]. Cooling temperatures activate these channels resulting in depolarization of TRPM8-expressing primary afferent neurons, which subsequently leads to the generation of sensory impulses [16]. Somatosensory behavioral responses to cold stimuli were partially deficient in animals in which TRPM8 was genetically deleted [13-15] but was almost completely deficit in animals whose TRPM8-expressing neurons were genetically ablated [17]. The potential involvement of TRPM8 in somatosensory behavioral hypersensitivity to cooling temperatures has been suggested in studies using animal models with chronic somatosensory nerve injury [14,18]. Consistently, inhibition of TRPM8 with capsazepine was shown to alleviate somatosensory behavioral hypersensitivity to cold stimuli [18].

Orofacial regions are innervated by the trigeminal nervous system, the cranial sensory system that shares many similarities with the somatosensory nervous system. However, differing from somatosensory nerves such as the sciatic nerve, the infraorbital nerve (ION) of the trigeminal nervous system is exclusively sensory and innervates the orofacial region most commonly affected in trigeminal neuralgia. Thus, studies on trigeminal neuropathy should be directly performed using trigeminal nerve injury models [19] although studies in the somatosensory system have provided useful information. Behavioral tests for cold sensitivity have been performed in orofacial areas previously, and most previous studies used the acetone spray method or similar paradigms [20]. However, these classical methods only test unlearned behaviors that are reflexive responses mediated by spinal cord and brainstem [6]. Reflex behaviors are not necessarily measures of pain induced by cold since they do not provide information on a higher order cerebral function [6,21]. Previous orofacial behavioral tests also have technical flaws, including stress of animals in restrained condition, anticipation of stimulation as animals can visualize probes approaching to them, and investigator bias. Therefore, it is essential to use a suitable behavioral assessment paradigm in order to clearly understand the mechanisms underlying the abnormal sensitivity to cold stimuli in animals with trigeminal neuropathy [19,22].

The orofacial operant test developed recently has overcome many problems of classical orofacial behavioral tests [23]. This new behavioral testing method uses a conflict paradigm to allow animals to make a choice between receiving a reward (drinking sweetened milk) or escaping aversive stimuli [23], and animals have control over the amount of nociceptive stimulation and can modify their behavior based on cerebral cortical processing [24,25]. Orofacial operant tests have been applied to examine physiological pain in response to noxious heat or cold stimulation in normal rats [26,27], orofacial inflammatory pain [23,28], and more recently for trigeminal neuropathic pain induced by chronic constrictive nerve injury of the infraorbital nerve (ION-CCI) [19,22]. In the present study, we applied the orofacial operant test to ION-CCI rats and determined effects of menthol and capsazepine, two compounds that can activate and inhibit the TRPM8 channel respectively, on operant behavioral responses to orofacial cold stimuli.

## Results

**Figure 1:**
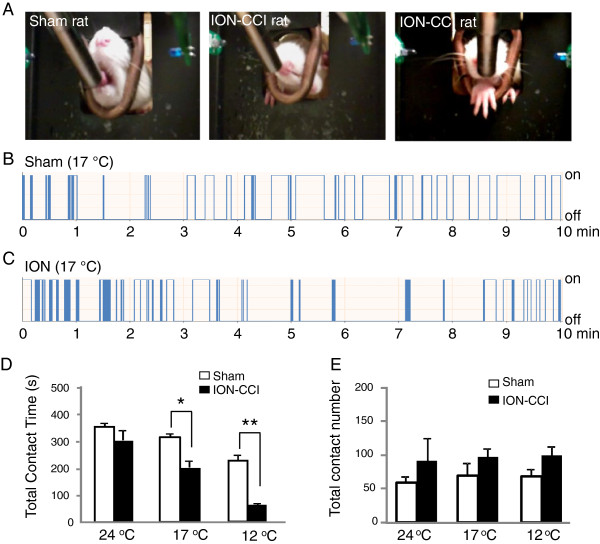
**Orofacial operant behavioral outcomes at cooling temperatures in sham and ION-CCI rats. A**) Left image shows that a sham rat was drinking milk while its cheeks contacted the thermal module. Middle and right images show that an ION-CCI (chronic constrictive nerve-injury induced by infraorbital nerve ligation) rat was biting the thermal module (middle) or reaching the nipple of the milk bottle with its front paw. The thermal module was set at 17 °C. **B**&**C**) Two sample traces show the automatic recordings of drinking behavior in a testing period of 10 min in a sham rat (**B**) and an ION-CCI rat (**C**). The thermal module was set at 17 °C in both **B** and **C**. Orofacial contacts were indicated by “on” and withdraws indicated by “off”. **D**&**E**) Summary of total contact time (**D**) and total contact number (**E**) in sham (open bars, n=7) and ION-CCI group (solid bars, n=7). The orofacial operant tests were performed with thermal module set at 24 °C, 17 °C, and 12 °C. Rats were tested during the postoperative period of 2 weeks to 6 weeks. Data represent Mean ± SEM; * *p*<0.05; ** *p*<0.01.

Orofacial operant tests were performed in both sham and ION-CCI groups to determine if chronic trigeminal nerve injury resulted in behavioral hypersensitivity to cooling temperatures. The thermal module was set at 24°C (neutral temperature), 17°C (cooling temperature), or 12°C (noxious cold). Figure 1A illustrates some noticeable differences in orofacial operant behaviors between sham (Figure 1A left) and ION-CCI rats (Figure 1A middle and right) at the cooling temperature of 17°C. Sham rats could contact the cooling thermal module with their cheeks for a long period of time while they were licking the nipple of the milk bottle (Figure 1A left, Figure 1B). In contrast, ION-CCI rats avoided prolonged contacts to the cooling thermal module with their cheeks, and the duration of each contact was usually brief at 17°C (Figure 1C). Sometimes, ION-CCI rats tried to bite off the thermal module with their teeth (Figure 1A middle) or tried to use their front paws to obtain milk from the nipple of the drinking bottle (Figure 1A right), but they gave up these behaviors shortly after a few attempts. Figure 1D compared total contact time between sham and ION-CCI groups at different temperatures. There was no significant difference in total contact time between sham group (352±10.3 s, n=7) and ION-CCI group (301.5±41.4 s, n=7) at 24°C. However, the total contact time was significantly shorter in ION-CCI rats (204.9±27.2 s, n=7) than in sham rats (313.4±11.4 s, n=7, p<0.05) at 17°C. At 12°C, the total contact time was also significantly shorter in ION-CCI rats (61.2±4.1 s, n=7) than in sham rats (229.6±20.7 s, n=7, p<0.01). We did not observe a significant difference in total contact number between these two groups at the three testing temperatures.

**Figure 2:**
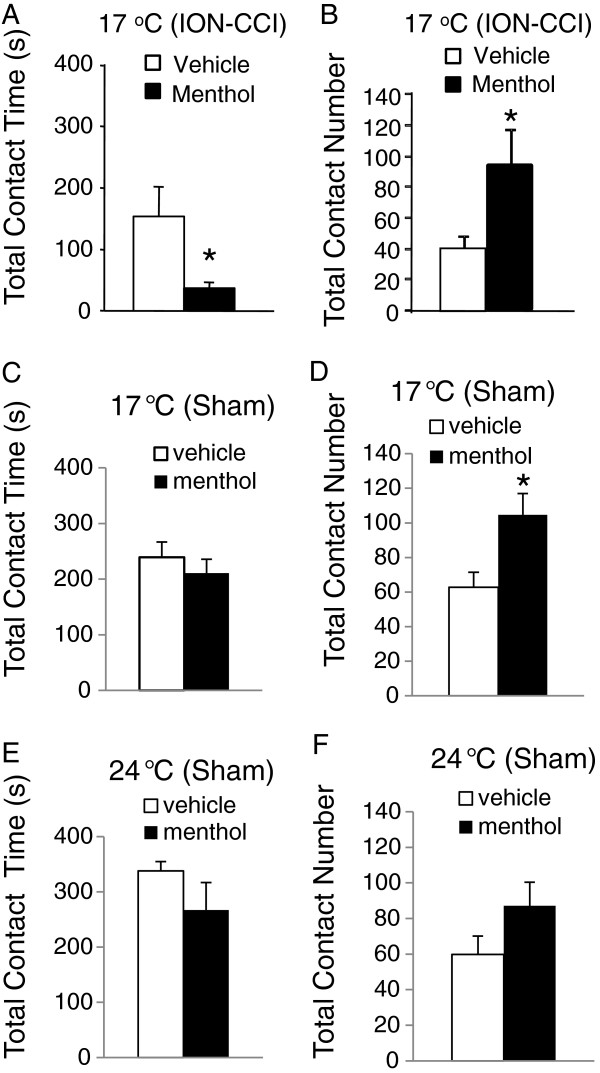
**Modification of orofacial operant behaviors by menthol at cooling temperatures. A**) Total contact time in ION-CCI rats following the administration of vehicle (open bar, n=7) and menthol (solid bar, n=7). **B**) Total contact number in ION-CCI rats following the administration of vehicle (open bar, n=7) and menthol (solid bar, n=7). **C**) Total contact time in sham rats following the administration of vehicle (open bar, n=7) and menthol (solid bar, n=7). **D**) Total contact time in sham rats following the administration of vehicle (open bar, n=7) and menthol (solid bar, n=7). Thermal module was set at 17 °C in **A** to **D**. **E**&**F**) Same as **C**&**D** respectively except thermal module was set at 24 °C. Data represent Mean ± SEM; * *p*<0.05.

We determined effects of menthol on orofacial operant behaviors in ION-CCI rats to see if cold hypersensitivity could be further exacerbated by the TRPM8 agonist. Menthol (10%) or vehicle was delivered by subcutaneous injection (150 μl) into the cheeks of the testing animals; the dose and route of menthol administration were based on a previous study in normal rats [27]. In comparison with vehicle control, menthol administration to orofacial regions of ION-CCI rats resulted in a significant reduction of total contact time (Figure 2A) in orofacial operant tests at 17°C; the total contact time was 153.7±48.4 s (n=7) with vehicle injection and reduced to 37.7±9.4 s (n=7) with menthol injection (Figure 2A). Total contact number was examined and ION-CCI rats showed significantly higher total contact number following the administration of menthol (94.3±26.7, n=7) than that following vehicle injection (40.0±8.9, n=7, P<0.05) (Figure 2B).

We determined effects of menthol on orofacial operant behaviors in sham rats to see if the TRPM8 agonist could sensitize cold response in rats whose trigeminal nerves were not injured. In comparison with vehicle control, menthol administration did not significantly affect the total contact time (Figure 2C) in orofacial operant tests at 17°C; the total contact time was 201.5±25.3 s (n = 7) with menthol injection and 239.1±27.5 s (n=7) with vehicle injection (Figure 2C). However, total contact number was significantly higher with menthol injection (104.8±12.2, n=8) than with vehicle injection (62.8±8.7, n=8, P<0.05) (Figure 2D). When orofacial operant test was performed at 24°C in sham group, neither total contact time nor total contact number was significantly different between menthol-injected group and vehicle control (Figure 2 E&F). The total contact time was 338.0±16.7 s (n=7) with vehicle injection and 267.0±33.1 s (n=7) with menthol injection; the total contact number was 59.9±10.3 (n=7) with vehicle injection and 87.1±13.3 (n=7) with menthol injection.

**Figure 3:**
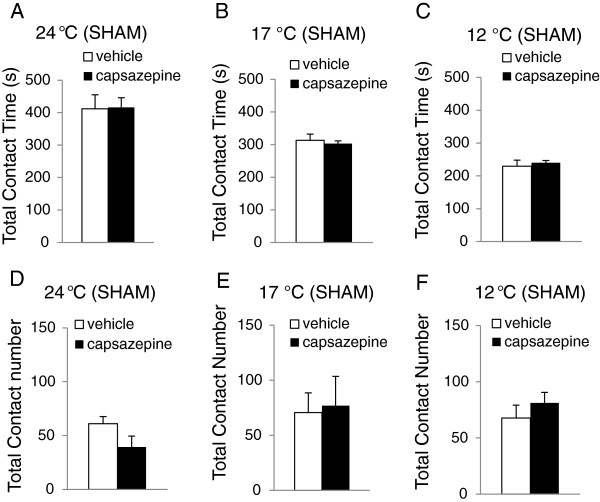
**Lack of effect of capsazepine on orofacial operant behaviors in sham rats. A**-**C**) Total contact time of orofacial operant tests in sham rats at 24 °C (A, n=5), 17 °C (B, n=5), and 12 °C (**C**, n=5). **D**-**F**) Total contact number of orofacial operant tests in sham rats at 24 °C (**D**, n=5), 17 °C (**E**, n=5), and 12 °C (**F**, n=5). Open bars, vehicle injection; solid bars, capsazepine injection. Data represent Mean ± SEM.

We examined effects of capsazepine on orofacial operant behaviors in sham rats to see if the TRPM8 antagonist could modify basal sensitivity to cooling temperatures in orofacial areas of these rats. Capsazepine at the dose of 3mg/kg was tested since our previous study showed that the cold hypersensitivity in the hindpaws of sciatic-CCI rats could be alleviated at this dose of capsazepine [18]. In comparison with vehicle injection, administration of capsazepine in sham animals did not significantly affect total contact time at 24°C (Figure 3A), 17°C (Figure 3B), and 12°C (Figure 3C). At 24°C, total contact time was 416.5±29.5 s (n=5) in capsazepine-injected group and 411.9±42.8 s (n=5) in vehicle control. At 17°C, total contact time was 303.3±8.3 s (n=5) in capsazepine-injected group and 313.4±18.4 s (n=5) in vehicle-injected group. At 12°C, total contact time was 240.1±6.8 s (n=5) in capsazepine-injected group and 229.6±18.5 s (n=5) in vehicle control group. Administration of capsazepine (3 mg/kg) in sham animals also did not significantly affect total contact number at 24°C (Figure 3D, 39.4±10.2 s, n=5 for capsazepine *vs* 61.0±6.6 s, n=5 for vehicle), 17°C (Figure 3E, 77.0±26.5 s, n=5 for capsazepine *vs* 70.6±17.9 s, n=5 for vehicle), and 12°C (Figure 3D, 81.2±9.3 s, n=5 for capsazepine *vs* 67.8±11.4 s, n = 5 for vehicle).

**Figure 4:**
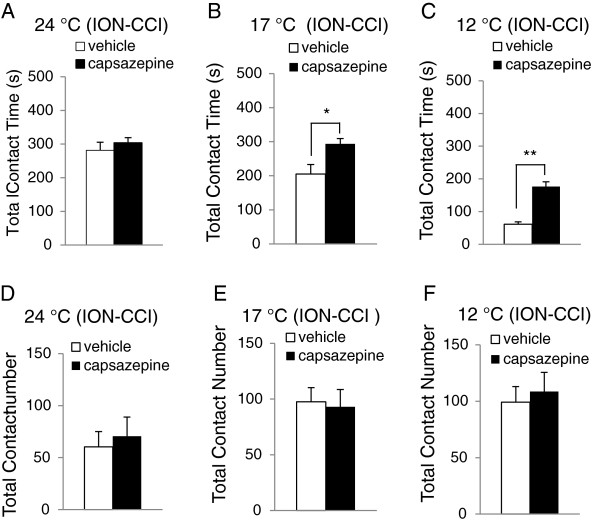
**Effects of capsazepine on orofacial operant behaviors at cooling temperatures in ION-CCI rats. A**-**C**) Total contact time of orofacial operant tests in ION-CCI rats at 24 °C (**A**, n=7), 17 °C (**B**, n=6), and 12 °C (**C**, n=6). **D**-**F**) Total contact number of orofacial operant tests in ION-CCI at 24 °C (**D**, n=7), 17 °C (**E**, n=6), and 12 °C (**F**, n=6). Open bars, vehicle injection; solid bars, capsazepine injection. Data represent Mean ± SEM; * *p* < 0.05; ** *p* < 0.01.

**Figure 5:**
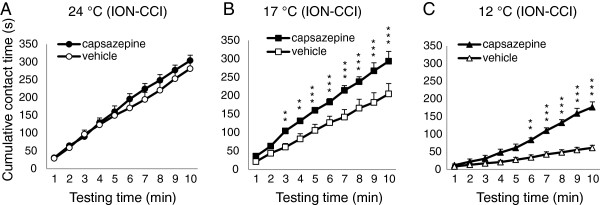
**Cumulative contact time of orofacial operant tests in ION-CCI rats following administration of capsazepine. A**) Cumulative contact time of orofacial operant tests in ION-CCI rats at 24 °C (n=7). Open circles, vehicle injection; closed circles, capsazepine injection. **B**) Cumulative contact time of orofacial operant tests in ION-CCI rats at 17 °C (n=7). Open squares, vehicle injection; closed squares capsazepine injection. **C**) Cumulative contact time of orofacial operant tests in ION-CCI rats at 12 °C (n=7). Open triangles, vehicle injection; closed triangles, capsazepine injection. Data represent Mean ± SEM; ** *p* < 0.01; *** p<0,001.

We then tested effects of the same dose of capsazepine (3 mg/kg) on orofacial operant behaviors in ION-CCI rats to see if cold hypersensitivity of these chronic trigeminal nerve-injured rats could be alleviated by the TRPM8 antagonist. At 24°C, total contact time was comparable between capsazepine-injected group (304.7±24.3 s, n=7) and vehicle injection group (281.4±24.3 s, n=7) (Figure 4A). However, total contact time was significantly longer in capsazepine-injected group than in vehicle-injected group at 17°C or 12°C. At 17°C, the total contact time was 205.0±28.2 s (n=6) in vehicle-injected group and increased to 293.6±15.5 s (n=6) in capsazepine-injected group (Figure 4B, P <0.05). At 12°C, the total contact time was 61.2±7.5 s (n=6) in vehicle-injected group and increased to 108.7±16.8 s (n=6) in capsazepine-injected group (Figure 4C, P<0.01). While total contact time was increased by capsazepine in ION-CCI rats, total contact number in these animals was not significantly different between capsazepine-injected group and vehicle-injected group at each temperature tested. At 24°C, total contact number was 70.6±18.4 (n=7) in capsazepine-injected group and 60.3±14.6 (n=7) in vehicle-injected group (Figure 4D). At 17°C, total contact number was 93.0±15.5 (n=6) in capsazepine-injected group and 97.5±12.7 (n=6) in vehicle-injected group (Figure 4E). At 12°C, total contact number was 108.7±16.8 (n=6) in capsazepine-injected group and 99.2±13.8 (n=6) in vehicle-injected group (Figure 4F). Because total contact time but not total contact number was modified by capsazepine in ION-CCI rats, we further examined cumulative contact time during the course of 10-min orofacial operant tests (Figure 5). At 24°C, capsazepine-injected rats and vehicle control had similar cumulative contact time at each time point (Figure 5A, n=7). In contrast, at both 17°C (Figure 5B, n=7) and 12°C (Figure 5C, n=7), cumulative contact time at most time points was significantly longer in capsazepine-injected group than in vehicle control group.

## Discussion

The animals’ orofacial operant behaviors are the integrative responses to sensory stimuli and cortical processing rather than simple noxious reflexes seen in the classical approaches (e.g. acetone spray method) for pain behavioral assessments [6,23]. We and others have recently used the orofacial operant test to reveal chronic orofacial neuropathic pain that was manifested by mechanical allodynia, cold allodynia, and cold hyperalgesia in the ION-CCI rats [19,22]. These symptoms well resemble the main symptoms of chronic trigeminal neuropathic pain in human patients. Thus, orofacial operant test on ION-CCI rats is a desirable approach in the present study to investigate pain-related behavioral outcomes following the activation or inhibition of TRPM8 channels on trigeminal afferent fibers.

Similar to our previous study, this study shows the presence of behavioral hypersensitivity to cold stimuli in ION-CCI rats, as was evidenced by the shortening of total contact time in ION-CCI rats at cooling temperatures. The present study further showed that TRPM8 agonist menthol exacerbated cold hypersensitivity in ION-CCI rats. This was evidenced by the significant decrease of total contact time following menthol administration in ION-CCI rats at 17°C. We observed an increase in the total contact number in ION-CCI rats when they were injected with menthol and tested at 17°C. This behavioral modification may represent a way for the ION-CCI animals to cope with the conflict paradigm so that they can obtain some rewards with least amount of cold-induced pain for each attempt. In the sham group, we showed that menthol had no significant effects on total contact time but increased total contact number at 17°C. The increase of contact numbers but no change in contact time tested at 17°C in sham rats may indicate that at this temperature menthol was mildly noxious but this mild noxious stimulus could not prevent sham animals from obtaining the rewards. This result is consistent with a previous study that showed slight sensitization of cold responses by menthol in normal rats at 10°C [27]. We tested menthol on orofacial operant behaviors in ION-CCI rats at 17°C but not at 12°C. This was because that at 12°C cold stimulation almost maximally reduced the contact time in ION-CCI rats, which would make it insensitive to observe a further exacerbation by menthol.

In contrast to menthol, capsazepine was shown in the present study to increase total contact time in ION-CCI rats at 17°C and 12°C. This suggests that cold allodynia/hyperalgesia was alleviated by the TRPM8 inhibitor in ION-CCI rats. This finding supports the idea that TRPM8 is involved in trigeminal neuropathy manifested with cold allodynia/hyperalgesia. The present study on trigeminal nerve-injured animals is consistent with our previous study showing that capsazepine could alleviate cold allodynia/hyperalgesia in sciatic nerve-CCI model of neuropathic pain [18]. It is known that capsazepine also inhibits TRPV1 [29]. However, the behavioral effects of capsazepine on cold sensitivity observed in the present study were unlikely to be due to its effect on TRPV1 because TRPV1 activation starts at ~43°C [30]. While capsazepine modified orofacial operant behaviors in ION-CCI rats, it did not significantly modify orofacial operant behaviors in sham animals tested at the cooling temperatures of 17°C and 12°C. This outcome may be due the incomplete inhibition of TRPM8 by capsazepine and the involvement in cold-transduction of other molecules in primary afferent fibers [9-12]. In fact, cold-sensitivity was only partially attenuated in mice whose TRPM8 channels were genetically deleted [13-15]. Interestingly, cold-sensitivity became almost completely deficit in mice in which whole TRPM8-expressing afferent neurons were ablated genetically [17]. Thus, cold-sensitivity appears to be not simply determined by a single transducer under physiological conditions.

In our previous study using CCI rats with sciatic nerve ligation, we have shown that the up-regulation of TRM8 in L5 DRG neurons is a molecular mechanism of the cold allodynia in sciatic nerve-CCI rats [18]. A recent study indicated that chronic trigeminal nerve injury also could result in the up-regulation of TRPM8 channels in trigeminal neurons and this change was accompanied by behavioral cold allodynia in orofacial area [22]. These findings are in line with our orofacial behavioral outcomes with menthol and capsazepine in IOC-CCI rats. However, since other sensory molecules are also involved in the transduction and conduction of cold stimuli [9-12,31], mechanisms underlying cold allodynia and hyperalgesia following chronic trigeminal nerve injury can be more complicated than what we have recognized at present time.

## Methods

Male Sprague–Dawley rats (300–450 g) were used in this study. All animals were exposed to light 12 hours per day; food and water were available ad libitum except where indicated. The protocol for the maintenance and use of the experimental animals was approved by the Laboratory Animal Medical Services and Institutional Animal Care and Use Committee at the University of Cincinnati and were in accordance with the NIH regulations on animal use.

Animals initially underwent two weeks of pre-surgical adaptation training utilizing the Ugo Basile Orofacial Stimulation Test System® (Comerio VA, Italy) after a 12-hour food fasting period. Rats were placed in a standard rat cage with a plastic divider to create two rooms, the testing room and the companion room. The presence of a companion expedited the adaptation of the rat being tested and promoted a consistent operant behavior. In the anterior aspect of the cage there was an Ugo Basile apparatus with a drinking window for the rat head to enter and acquire a reward (milk). Nestle Carnation® Sweetened Condensed Milk was diluted with deionized water to 30% and placed in a cylindrical plastic container with metal nipple drinker. The apparatus also consisted of a thermal module but the module was removed during adaptation training period. An infrared photo-beam was built on the exterior aspect of the drinking window and wired to a computer to automatically detect the head accessing the feeding tube. The training was started by placing a rat in the testing room and another one in the companion room. After the rats were given 10 minutes to familiarize themselves with their environment, the drinking window was opened and the testing rat was subsequently timed for 10 minutes to allow drinking the milk.

After 2 weeks of the pre-surgical adaptation training, the rats were divided into two groups: sham and ION-CCI (Infraorbital nerve ligation). In the ION-CCI group a chronic constriction nerve injury model was created using unilateral ligation of the infraorbital nerve as described previously [19,32] In brief, the rats were anesthetized with intraperitoneal injection of ketamine/xylazine cocktail (100 mg/kg). The skin above the right eye was shaved and the rat head was immobilized. A 2-cm curvilinear incision was made superior to the right orbital cavity. A meticulous dissection was made, and the muscle and fascia were retracted laterally. The infraorbital nerve can be found approximately 1 cm down against the floor of the maxillary bone. The nerve was freed from the surrounding connective tissues and two ligatures were made approximately 5 mm apart with a 5–0 absorbable chromic gut suture Superion® [19]. The incision was closed with 6–0 non-absorbable braided silk suture. The sham groups also had a similar surgery, but without any ligatures. The nerve was freed from the surrounding connective tissue and the incision closed. After a 2-week healing period, the rats underwent a 2-week period of post-surgical adaption training performed in the same manner as the pre-surgical adaptation training.

Subsequently, experiments were performed utilizing the thermal module during post-operative period of 2 to 7 weeks, a period in which ION-CCI rats consistently showed cold allodynia and hyperalgesia in the orofacial operant tests [19]. In the thermal module there was a surrounding metal tubing at the opening filled with circulating ethylene glycol (Sigma Aldrich, US) made in a 50/50 mixture with distilled water. The temperature of the circulating ethylene glycol solution was controlled by a thermal circulating bath unit. The distance between the metal tube and the nipple of the milk bottle was 14 mm. For thermal stimulation, thermal module was set at 24°C, 17°C, or 12°C. In order to create an orofacial region that is more sensitive to cooling temperatures, the sham and ION-CCI group rats’ facial areas were shaved one day before orofacial operant testing. The orofacial region is innervated by the infraorbital nerve from the V2 branch of trigeminal nerve. The ligation of infraorbital nerve produces nerve injury which affects the sensations of orofacial region to thermal stimuli when the rat contacted the metal tube as it poked its head through the hole to obtain the milk.

To test the effects of menthol on cold sensitivity in ION-CCI rats, menthol (10%, 150 μl) was injected ipsilaterally to the cheeks (S.C.) of the ION-CCI rats. Menthol ((1R, 2S, 5R)-(−)-Menthol, Sigma, St. Louis, MO, USA) solution was made with vehicle that contained 10% menthol, 1.6% ethanol, 1% Tween80, and 87.4% saline. To test the effects of capsazepine (3 mg/kg, S.C. into the backs of animals) on cold sensitivity of ION-CCI rats, capsazepine (Tocris) was made in vehicle that contained 20% dimethyl sulfoxide, 1% ethanol, 1% Tween, and 78% saline. The test compounds were administered 30 min prior to orofacial operant tests. On different days these animals were also administered vehicles as controls and then orofacial operant tests were performed in the same manner. Similar to the adaptation training, all experiments with thermal module were preceded by a 12-hour fasting period, 10 minutes for the rats to be familiarized with testing environment, and a subsequent 10 minutes to allow for orofacial operant behavioural assessment.

The events of head pokes were detected by the infrared photo-beam, recorded by a computer, and analysed by the Oro Software (Ugo Basile, Comerio VA, Italy). This computer software recorded and analysed several variables of the rat’s behaviour including the total time the beam was broken (also defined as the total contact time), and the total count, which can also be described as total contact number. In some cases, ION-CCI rats bit thermal module or used their front paws to reach the mild drinker. Biting thermal module did not make beam breaks because the infrared photo beam was far away from the thermal module. The use of front paws to obtain milk could sometimes make beam break. However, animals quickly gave up these aberrant behaviours after a few trials at the beginning the tests and these events were not removed from the event data. Unless otherwise indicated, total contact time and contact number in a 10-min experimental session were used as orofacial operant behavioural parameters. Data were presented as Mean ± SEM, analysed by the paired Student’s t test or two-way ANOVA with Bonferroni post test, * P < 0.05, **P<0.01, and ***<0.001.

## Competing interests

The authors declare that they have no competing interests.

## Authors’ contributions

XZ and JXL carried out orofaicial operant behavioral tests and data analysis. JGG designed experiments. JGG and GYX performed statistical analysis and drafted the manuscript. All authors were involved in reading, amending and approving the final manuscript.
